# Collectanea

**Published:** 1830-11

**Authors:** 


### COLLECTANEA.

**Floriferis ut apes in saltibus omnia libant,**
**Omnia nos, itidem, depascimur aurea dicta.**

### PRACTICAL MEDICINE.

*On the Severe and Fatal Consequences resulting from Slight Wounds in Dissection.* (From the *Glasgow Med. Journal.*)

An interesting article upon this important subject is contained in the August Number of the Glasgow Journal. We give the substance of the communication in the writer's language.

"The first inquiry I shall submit to investigation may be thus stated: Is there evidence sufficient to establish and confirm the following proposition? There is a difference in the form and in the severity of the consequences resulting from dissection wounds, according to the disease which may have produced death, and to the stage of decomposition.

"I submit, as premises from which an answer to this inquiry is to be deduced,

"1st. That I have perused and kept a list of above forty published cases of dissection wounds, and I find, from that list or table, that a very large majority occurred in the dissection of recently deceased bodies before interment. Out of the whole number, only two* had their origin in the dissection of putrid bodies, and in reference to which Dr. Duncan has observed, "All the cases which I have observed, or of which I have had accurate reports, except that of Mr. Whitelaw and No. 17,† occurred after the examination of recent bodies before they were interred."‡ Both of these cases recovered; in the former the disease was severe, in the latter rather slight.

"2d. The majority, in as great a proportion of cases, not published, which have come to my knowledge, has in like manner arisen from the exposure of a raw surface in contact with the fluids of a body recently deceased."

Dr. Colles is of opinion, from observation and reflection, that "putrefaction (meaning the state of putridity) gives protection to the anatomist."§

"From these premises, the conclusion, I think, is warrantable that, from inoculation with the fluids of a body recently deceased, more danger is to be apprehended than from a similar exposure to the fluids of a body further gone in decomposition."

A majority of the cases which have been published, have been affected after the examination of the bodies of persons who had died of some affection of the serous membranes.

"A majority, in as large a proportion of cases, not published, which have come to my knowledge through other channels, has originated in similar circumstances."

Mr. Shaw states|| that there is more danger from a wound received in examining the body of a patient who has died of peritoneal inflammation, than any other disease.

"Sir A. Cooper has narrated a case in the third volume of his Lectures, illustrative of the observation with which he prefaces it, and bearing upon the determination of the present question. His observation is thus expressed: 'It would seem that, under

* Nos. 9 and 16.
† No. 16 in my Table.
‡ Edin. Med. Chirurg. Transactions, vol. i.
§ Dublin Hospital Reports.
|| Medical and Physical Journal, May 1825, p. 373.

certain circumstances, a poison is produced, sufficiently strong to excite inflammation, even when there is no wound.'

"The case is then narrated. 'Mr. Cook, surgeon, at Marsh-gate, Westminster bridge, sent to me, whilst labouring under the highest irritative fever, in consequence of having opened the body of a person, who had died of puerperal fever. When I examined him, I found the extremities of his fingers of both hands inflamed, as if they had been dipped in scalding water, and the absorbents of his arms red, hard, and knotted, to the axilla; yet he had not any wound or abrasion of any kind upon his hands, and it would, therefore, seem that the fluid produced in the abdomen of this woman, in which his fingers had been frequently immersed, was of a highly stimulating nature.'

"M. Begin has narrated a somewhat similar case, but I have been unable to procure a detail of the particulars.

"These circumstances, especially the case of Mr. Cook, seem to me to prove, incontestably, that from some bodies more danger is to be dreaded than from others, and that those which present serous effusions are such."

Having endeavoured to determine the character of the exciting causes, the author proceeds to the investigation of those conditions of the system which may be denominated predisposing causes.

"Upon this topic, it may be inquired, Are there any such conditions, that, by rendering the body more susceptible of the impression of the excitants, or by modifying the consequences of wounds in dissection, can be justly entitled to rank under the denomination of predisposing causes? And, what are these conditions?"

On these questions different opinions have been entertained: while some attribute to a certain bad habit of body the whole *origo mali*, others affirm that the state of the constitution has no share in the production of the evil at all. Various authorities are referred to, and the inference appears to be established,

"That to certain conditions of the system may be attributed the power of rendering us more susceptible of the severe consequences from dissection wounds; and that these conditions are such as are usually expressed by the rather vague appellations of 'irritable or bad habit of body,' 'scrofulous diathesis,' 'worn out constitution,' 'general debility,' &c. names for a condition, of which, in general, no very precise ideas are formed."

The paper concludes with a few observations on the treatment of the consequences of dissection wounds.

"I may presume upon the reader's acquaintance with the most important of the practices which have been adopted. Sir A. Cooper's sentiments on the treatment may be found in Butter on Irritative Fever; Mr. Shaw's, in the London Med. and Phys. Journ. May 1825; Dr. A. T. Thomson's, in Lond. Med. Repository, April 1825; Dr. Goodman's, in Philad. Journal, No. 18; Mr. Higginbottom's, in his Treatise on Lunar Caustic; Mr. Lawrence's, in London Med. Gaz. vol. v. And I may also presume upon his acquiescence in the following judgment upon all the plans hitherto tried, pronounced by Dr. Colles. 'Whatever difference of opinion,' says he, 'may be entertained as to the nature of this affection, it will be allowed on all hands, that, although some few have escaped, yet the plans of treatment hitherto pursued have all proved quite unequal to contend with so formidable a disease."*

Dr. Colles proposes a new plan, to administer calomel with the view of speedily

* Dublin Hospital Reports, vol. iv.

exciting ptyalism. He recommends three-grain doses every three or four hours, uncombined with opium or any other medicine.*

"This was published about the middle of the year 1827, and, so far as I know, no case has been published since, either by Dr. C. or any other person in which this suggestion was put to a trial. In the month of January 1829, I suffered from the exposure of a raw surface during dissection, and having tried this mode of treatment proposed by Dr. C., I shall take this opportunity of relating the case.

"About the end of January 1829, I assisted at the inspection of the body of a female who had died of chronic pulmonary disease, with effusion betwixt the pleuræ on the left side. While handling the lung on this side, I felt a smarting pain in a scratch upon my right thumb, which I had, I thought, sufficiently cauterized with the nitrate of silver, before going to the inspection. I immediately washed and sucked the wound, and re-applied the caustic. This was about four P.M.

"2d day. About five A.M. next morning, that is, thirteen hours after the inspection, I was awakened by a severe pain above my elbow, and on the top of my arm, in the situation of the deltoid muscle. This pain increased in severity till eight A.M. when I commenced the use of a cold lotion to the arm, and calomel in three-grain doses every three hours. Had slight headach and languor. Pulse eighty-two.

"3d day. After taking five doses of the calomel it was discontinued, as by that time it had begun to act on the bowels. During the night it acted four times very freely. Sleep very disturbed. In the morning I felt very languid, but scarcely any pain in the arm. Tongue very foul. Pulse 96 to 108. Nausea prevented me from living according to Mr. Shaw's stimulating plan of regimen, which I had resolved to do. In the afternoon I began to feel my mouth a little sore, and about five P.M. on attempting to chew, felt my gums very sore, and when looked at they were found very red, with a few white spots upon them. The languor now went off. Later in the evening I felt the gluteal region of the right side very tender on pressure. The elbow and shoulder not painful except on pressure or motion.

"4th day I felt so well as to be able to resume my professional visits. After walking four miles, I felt languid and irritable, and the pain returned in lumbar and gluteal regions. After using my arm a little, it also became very painful. Re-applied cold lotion. Gums not very sore. No salivation. Pulse 92 to 100.

"5th day. During this day felt spasmodic twitching in pectoral region. The pain in lumbar and gluteal regions rather increased. Pulse ninety-two. Tongue furred and white. Tenderness of mouth and gums nearly gone. Pain in arm very little until I had used it a little.

"6th day. About midnight the pain of arm became excessive, and more nearly resembled the sensation of what is called a sleeping foot or hand, than any other I can remember, in kind, but not in degree. It continued very painful for eight hours, notwithstanding the use of the cold lotion a part of that time, and taking ʒi. of Tinct. Opii. About one A.M. recommenced the use of the calomel, of which I again took five doses. In the afternoon my mouth became sore, the calomel not having yet acted on the bowels, and in the evening I felt completely relieved from all my complaints, local and general.

"7th day. Slept well. Mouth very sore and blistered. Arm nearly free of pain even on pressure. Some red spots were observed on shoulder today. No swelling in axilla. Yesterday one of the veins of the arm, a branch of which ran over the

* Dublin Hospital Reports, vol. iv. p. 249.

thumb, was observed tense, hard, and painful. Today the pain and hardness are gone.

"8th day. General irritation less. Pulse ninety-two. Tongue white but cleaning. Arm a little painful. Mouth less sore.

"9th and 10th days. Arm a little painful. Mouth getting better.

"11th day. Yesterday evening my mouth again became painful, and the gums swollen and blistered, in consequence, I suppose, of being out part of the day, which was damp and foggy. This day felt less pain, and was less oppressed or languid than on any previous day. Commenced the use of Sulph. Quin.

"From this day I was free from complaint, save of weakness, and of the arm being very easily fatigued.

"The most remarkable circumstance in the history of this case certainly is, that the symptoms, both local and constitutional, were thrice checked in their progress at the time of the mercurial action being most sensibly felt. And if not the consequence of the mercurial action, of what was this check the consequence?

"This case, as I said before, is the only one, so far as I know, in which mercury has been used with the view of obtaining its specific effect; and among all the histories of similar cases which have been published, or privately related to me, (upwards of fifty in all,) it is not mentioned that in any, save one, was the specific effect of mercury produced by what of it had been administered for other purposes. The particulars of this case have been given to the world by Mr. Shaw.* In this case also, so soon as the mouth was affected, all the symptoms, local and constitutional, disappeared. Moreover, in other respects, it is a very interesting case.

"Though these cases cannot be considered as evidence sufficient to establish the certain efficacy of this mode of treatment, they seem to hold forth abundant inducement to make further trials, and to claim for this treatment a confidence, equal, if not superior, to that which can be reposed in any other. It is not simply and singly because the cases terminated favorably, that I would attach more confidence to this, the mercurial, than to any other mode of treatment; but it is, because the subsidence of the symptoms and the appearances of mercurial action; the annihilation, or at least the overpowering of the natural by the artificial disease, appear so intimately related by the simultaneousness of their occurrence, as to lend considerable plausibility to the hypothesis that the relation was, not merely that of casual coincidence, but that of cause and effect."

---

*Plague cured by Turpentine and Camphor.* (Dr. M'Cabe *on Fever. Midland Med. and Surg. Reporter.*)

In the treatment of fever, and, indeed, in the treatment of all diseases, our practice ought, if possible, to be guided by certain and fixed principles. The practitioner who is constantly changing his ground and changing his remedies, is not likely to be a successful one; an experimenter in medicine is a dangerous philosopher. If chance or accident, however, enables us to add to the catalogue of remedies for any disease, a new or more powerful one, or one by which general principles may be better explained or more fully elucidated, we should avail ourselves of the opportunity.

In Sir Arthur Brooke Faulkner's able work on the plague, which occurred in Malta in 1813, he has published the details of nine cases of that formidable disease, which were treated under his direction and care in the pest hospital of that island. Out of

* London Med. and Phys. Journ. 57, 142.

the nine published cases, I find that six proved fatal; and of the three successful ones, I find, by his notes, that a large quantity of spirits of turpentine and camphor, which was intended for an external application to carbuncles, had been given internally by mistake in two of them, and had occasioned a severe hypercatharsis. The following are the words in which Sir Arthur noticed the circumstances in his case book: "On looking accidentally into the hospital-mate's prescription book, I observed that one drachm of camphor, and four ounces of oil of turpentine, had been directed for the carbuncle, which, upon inquiry, I found the attendants had administered internally! This will fully account for the purging on the 30th and 31st." This case recovered; the other is mentioned in the following words:

"Die 31 (Julii 1813). Most severely purged in the night. On looking into the prescription book of the hospital-mate, I observed the same application for the carbuncle as ordered for Yaniwicz above; viz. four ounces of oil of turpentine, and one drachm of camphor. Vespere. Continues still much purged, and complains of great griping and uneasiness about the belly.

"Die Augusti 1. I found the same mistake had been committed as in the case of Yaniwicz, and the oil of turpentine and camphor had been given internally." This patient also recovered.

Sir Arthur Brooke Faulkner does not appear, in what he has published on the subject of plague, to have drawn any practical conclusions from the remarkable facts which I have here given from his book. That of the three recoveries out of nine cases, two of them should have taken such powerful purgative and stimulant medicines by mistake, appears to me too remarkable a circumstance to be considered merely a coincidence. The inference appears to be that their recovery was owing to the mistake. Although the inference may be admitted, few would choose to give four ounces of spirit of turpentine and a drachm of camphor as a stimulating purgative, although it was successful in these cases: perhaps strong drastic purgatives, such as colocynth, scammony and calomel, with diffusible stimuli, such as æther, camphor, opium and wine, would have answered the purpose, and might be considered the safer; but the great principle which I think these remarkable facts go far to establish, is, that purgation and stimulation are the appropriate remedies for the plague.

The application of these observations to fever, must be obvious. Plague is probably nothing more than a form or variety of fever; violent and destructive in proportion to the intensity of the case. The yellow fever of the West Indies, and of the shores of the Mediterranean, during the prevalence of an epidemic, is often as destructive in its ravages, and as rapid in its progress, as the plague. Of the plague, I have no knowledge from personal observation; but of the yellow fever, I have had much experience, and the practice which was found by far the most successful in the treatment of it, consisted of purgation and the antiphlogistic regimen in the early stages, taking care to prevent too much exhaustion, by the occasional exhibition of wine, camphor, and other diffusible stimuli, particularly in the latter stages of the disease; and after some experience of the typhous fever of this climate, I feel convinced that the most successful practice in it also is that which is guided by the same principles.

---

### SURGERY.

*Lithotomy.* The following case, which lately occurred in my private practice, seems deserving of notice, as being very unusual in several respects. A few weeks ago, Dr. T. Thomson asked me to see a boy ten years of age, the son of an artist in town. He had been complaining for five years of the usual symptoms of stone, which latterly confined him to the house in the greatest misery. He had been passing small irregular fragments of calcareous matter.

On attempting to introduce a sound proportioned to the usual size of the urethra at his age, I met with an obstruction about three inches from the orifice, which required a good deal of pressure to admit the entrance of a small instrument. I felt a calculus lying in the neck of the bladder, or rather anterior to it; and, putting my finger into the rectum, ascertained that this was really its situation. I performed the operation next day in the ordinary way, and extracted a mass of calculous matter the size of a walnut, which seemed to have originally consisted of two nearly equal concretions: one of these was entire, and presented a smooth flattened surface to the other, the external shell of which was broken into fragments similar to those voided previously to the operation. I then examined the bladder by a sound, and ascertained that it contained no other calculus.

Every thing went on favorably until the urine ceased to flow through the wound, which happened about the end of a fortnight, when he began to complain very nearly as much as before. I concluded that the stricture must now be the cause of her sufferings, and proceeded to cure it by the ordinary process of dilatation. I am now able to pass number 4, and he is free from complaint. I never saw or heard of a stricture in the urethra in so early a period of life, and I rather suspect it was not a consequence of the irritation of the calculus, but the original disease, since his uneasy feelings were distinctly dated by his friends to an inflamatory affection of the penis, which occurred when he was five years of age.—From Mr. SYME's *Quarterly Reports, &c. Edinburgh Med. Journal.*

---

*Surgical Recovery of an Eye.* M. MAUNOIR, professor of surgery at Geneva, having performed the operation for cataract, by extraction, upon a man eighty-two years of age, weakened by an operation for hernia, which he had endured six weeks before, perceived to his regret that, although the pupil remained beautifully black and perfectly intact, the anterior and posterior chambers of the eye were not replenished, the cornea became sunk and wrinkled, a few bubbles of air penetrated the anterior chamber, and the patient had no vision. Without yielding to the first melancholy impression the operator, by a happy presence of mind, conceived the hopes of filling the cavity: he sent immediately for some distilled water, warmed it, placed the patient on his back and filled the external orbit of the eye with the water, opened the eyelid, and raised the flap of the cornea. The water then penetrated into all the accessible cavities, the folds of the cornea disappeared, and its convexity was restored. Having kept the eye shut for some minutes, he then directed the patient to open it, and found it in the most satisfactory condition; the patient distinguished all the objects presented to him as well as after the most successful operation. A slight pain was felt after the introduction of the water, which went off after a short time. From that time the eye healed without difficulty, and when opened a week after the operation it was free from swelling and inflammation; the cornea was perfectly united, but the pupil was a little obscure, the sight feeble, and the patient complained that he did not see so well as immediately after the operation. But, six days after the bandage was removed, the shade of the pupil was much diminished, the sight grew stronger from day to day, and no doubt was entertained that the patient would soon be able to read common print.—*Bib. Universelle. Journal of the Royal Institution.*

### CHEMISTRY.

*On the Pharmaceutical Preparation of the Precipitated Carbonate of Iron.* By Mr. Thomas Clark. (*Glasgow Med. Journal.*)

In order to prepare this medicine, the British Pharmacopœias direct that watery solutions of sulphate of iron, and of subcarbonate of soda, be mixed, and that the resulting precipitate should be collected by a filter and dried. The precipitate when it first appears, is white, but it soon becomes of a dark green; and when it subsides, it is found a soft pulpy mass, occupying a considerable bulk. Transferred to a filter, and there drained of its water, it is found to be little contracted in its bulk; and it still retains much water, like a sponge, in its pores. If we watch the surface which is exposed to the air, we may observe its dark green gradually change into a rust yellow. The change, it is known, is produced by the oxygen of the atmosphere. The precipitated carbonate of iron consists of carbonic acid combined with the black oxide of iron; which black oxide readily combines with more oxygen forming the red oxide; but, as the red oxide cannot, like the black oxide, retain carbonic acid in combination, this acid flies off; so that, in the yellow matter alluded to, an additional dose of oxygen has taken the place before held by carbonic acid. The yellow colour is owing to the red oxide existing in combination with water, or (to use the language of modern chemistry,) as a hydrate; and the yellow colour is changed to red whenever we apply so much heat as will drive off the combined water. Then the red oxide of iron, or colcothar of vitrol, alone remains.

This decomposition of the precipitated carbonate of iron by the action of the air might be disregarded, if it were confined to the surface; but it is far otherwise.

The bulky precipitate on the filter when it dries, whether by spontaneous evaporation, or by the aid of a gentle heat, contracts but little in its bulk, and consequently is left a light porous mass. Thus does the air obtain access to every particle of it; and thus does the decomposition, which we at first witness on the surface, penetrate inward. One, indeed, is apt to think that, as little air can get into the pores, little action can be produced in the interior, compared with what we observe on the surface. Much otherwise, however, will be the opinion of the chemical experimentalist of accurate observation, who knows that every porous substance absorbs air to a wonderful extent, (charcoal, for example, thirty and even fifty times its own bulk of some gases, and consequently many more times the bulk of its pores;) and who has been taught by the action of spongy platinum on mixed gases, that in their absorbed state gases evince an active tendency to combination, which they do not manifest under other circumstances.

Besides the almost unavoidable action of the air, the action of heat is almost always employed by the ignorant pharmacian to further the decomposition of the precipitated carbonate of iron; insomuch that, by these combined actions, the substance sold under this name hardly contains more than a trace of carbonate of iron, and not unfrequently is nothing else than colcothar of vitrol. Now, colcothar of vitrol is not less different from carbonate of iron in its medicinal than it is in its chemical properties. I have seen patients, of different ages and sexes, swallow, for a fortnight, at the rate of half an ounce a-day, of colcothar of vitrol, without producing any apparent effect, except that their stools were coloured by the powder, to a reddish hue, indicating that it had passed through the body unaltered; whereas I have seen a healthy man made sick by a dose of a quarter of a drachm of genuine carbonate of iron, and made to pass, in consequence, dark greenish black stools for two days after; and I have seen similar effects produced on patients who had been unaffected by colcothar of vitrol. The sickness, however, is not produced after the first or second day.

I call the attention of medical practitioners to these observations, and the more earnestly, that of late it has become fashionable to prescribe large doses of carbonate of iron in certain nervous diseases; and I hope that I shall perform no unacceptable service to those gentlemen, if, by concluding this paper with a simple process for obtaining carbonate of iron in a fit state for exhibition, I dissipate the uncertainty of its effects, by banishing uncertainty from its preparation, and thus enable them to repose on the evidence of their own observation; a practice which too much as yet, I fear, rests on deference to authority.

From the preceding observations, it is easy to gather, that the two defects to be avoided are exposure to air and exposure to heat. Both of these defects I propose to avoid, by forming the precipitated carbonate into an electuary: thus,

Take of sulphate of iron and subcarbonate of soda, each eight ounces. Pound each salt, and dissolve them separately in warm water. If necessary, filter. Being filtered and cool, mix the solutions in a deep vessel, capable of holding one or two gallons of water, which fill up cold. Stir, let subside, and then decant the clear liquor from the precipitate. Fill up again with water, and likewise again decant; and repeat this operation two or three times, so as to separate the soluble salts. Next put the precipitate on a filter of cotton or linen cloth, supported by a square frame. When the water has ceased to pass, gather into one hand the edges of the filter, so as to make it a sort of bag, and with the other twist it round from the holding hand downwards, so as to squeeze out the remaining water. The precipitate will now have the appearance of clay, too soft for moulding. With soft sugar and aromatic powder, in suitable proportions, make it into an electuary.

Thus we obtain a carbonate of iron, uniform in its properties, hardly deteriorated by the process it undergoes, and little liable to change by keeping.

The precipitated carbonate of iron, while yet moist, is soluble in carbonic acid. Hence a teaspoonful of the above electuary is soon dissolved in a glass of ginger beer, except the aromatic powder it contains. It may be asked, therefore, whether an eligible medicine might not be obtained as follows: Having filled a dozen of bottles with ginger beer, divide among them the precipitate from an ounce of sulphate of iron, and an ounce of subcarbonate of soda: then cork and set them aside, as usual, till they be ready. I presume that the production of carbonic acid, by the fermenting process, would go on as usual, and that when drawn in due time, we would find the carbonate of iron entirely dissolved in the ginger beer.

---

*Production of Hydrocyanic (Prussic) Acid under uncommon Circumstances.* Dr. A. Hayes. Wishing to decompose some nitric acid containing about one third its weight of dry acid, it was subjected to distillation with one third of its weight of a raw sugar; the distillation was attended by the production of vapors of nitrous and hyponitrous acids, as is usual in the decomposition of nitric acid. The fluid in the receiver was slightly acid, it was therefore returned to the retort still containing the residue of the first operation, and gentle heat applied; the strong and peculiar odour of hydrocyanic acid was developed, in such a quantity as to render the atmosphere of a small room irrespirable. After cooling the apparatus and decanting the distilled fluid, a few drops of ammonia were added, and the alkaline fluid, mixed with a solution of proto-sulphate of iron, and a few drops of acid, deposited a bulky precipitate, which, on exposure, became of a fine blue colour.—*Rosebury Laboratory, March* 16, 1830. *Silliman's Jour.*

*Salicine; its Power as a Febrifuge.* A very important Memoir by M. LEROUX, which was presented to the Academy of Sciences, has been most favorably reported upon by MM. GAY LUSSAC and MAGENDIE. It relates to nothing less than the discovery of a principle in indigenous plants which may replace quinia and cinchonia as medical remedies. Being aware that the willow had been employed advantageously as a bitter and febrifuge, M. LEROUX sought in it for some active principle, and ultimately sent two preparations to the Academy, one called salicine, the other sulphate of salicine. He at first thought the new principle was a vegeto-alkali, but when afterwards in Paris, he convinced himself that it had no power of neutralizing acids, did not combine with them, was rendered uncrystallizable by them, contained no nitrogen, and was not a vegeto-alkali. The sulphate was a mistake.

Salicine is in the form of very fine nacreous white crystals, very soluble in water and alcohol, but not in ether; it is very bitter, and partakes of the odour of willow bark. In order to obtain it, three pounds of the bark of the willow (Salix helix), dried and pulverized, is to be boiled in fifteen pounds of water, with four ounces of carbonate of potash, for an hour; it is to be filtered, and, when cold, two pounds of solution of sub-acetate of lead added; when settled, it is to be filtered, treated with sulphuric acid, the rest of the lead precipitated by sulphuretted hydrogen, the excess of acid neutralized by carbonate of lime, again filtered, the liquid concentrated and saturated by dilute sulphuric acid, then boiled with animal charcoal to remove colour, filtered hot, crystallized repeatedly, and dried without access of light. About one ounce of salicine will be obtained in the large way; probably twice the quantity would result, for great loss is occasioned by the above numerous operations. It may be preserved in well-closed bottles, and does not attract moisture.

As to the medicinal powers of this substance, M. Magendie states, that his own experience of its effects in intermitting fevers is favorable, and that he has seen three doses, of six grains each, stop a fever. He quotes the experiments of MM. Miquel, Husson, Bally, Girardin, Cognon, &c., at the hospitals and elsewhere, in its favor: they all agree in its anti-febrile power, and in stating that from twenty-four to thirty grains of salicine will arrest the return of the fever, whatever may be its kind. This is nearly the same as the dose of the sulphate of quinia.

In concluding, the commissioners state that M. Leroux has discovered in the willow (Salix helix), a crystallizable principle which approaches sulphate of quinia in its anti-febrile power, and that this discovery is, without contradiction, one of the most important that has been made for many years in pharmaceutical chemistry.—*Journal of the Royal Institution*, from the *Ann. de Chimie.*

